# Supplementary material for: Novel Alzheimer risk genes determine the microglia response to amyloid‐β but not to TAU pathology
Source: EMBO Mol Med. 2020 Jan 17;12(3):e10606. doi: 10.15252/emmm.201910606 (PMC7059012; doi:10.15252/emmm.201910606)
Supplement: Supplementary file 1 — Appendix [file EMMM-12-e10606-s001.pdf]

Appendix to Sierksma et al. 2019

Table of content

1. Appendix Figures:
    1. Appendix Fig.S1: Two APPtg-10M mice have lower transgene expression.
    2. Appendix Fig.S2: Overview of APPtg-based WGCNA modules.
    3. Appendix Fig.S3: Overview of TAUtg-based WGCNA modules.
    4. Appendix Fig.S4: Expression of myeloid marker gene sets across the UMAP of the single microglia sequencing data.
    5. Appendix Fig.S5: Division of experimental groups within each cluster
    6. Appendix Fig.S6: Examples of the mean z-score distribution of 10,000 randomly sampled gene sets with equal sizes to the gene set of interest.
2. Appendix Tables
    1. Appendix Table S1: Overview of the number of Marioni-based GWAS genes at different p-value cutoffs.
    2. Appendix Table S2: Overlap between genes within a WGCNA module and the cell-type specific gene sets.
    3. Appendix Table S3: Results from the SuperExactTest for overlaps between multiple gene sets.

Appendix Figures and Figure Legends

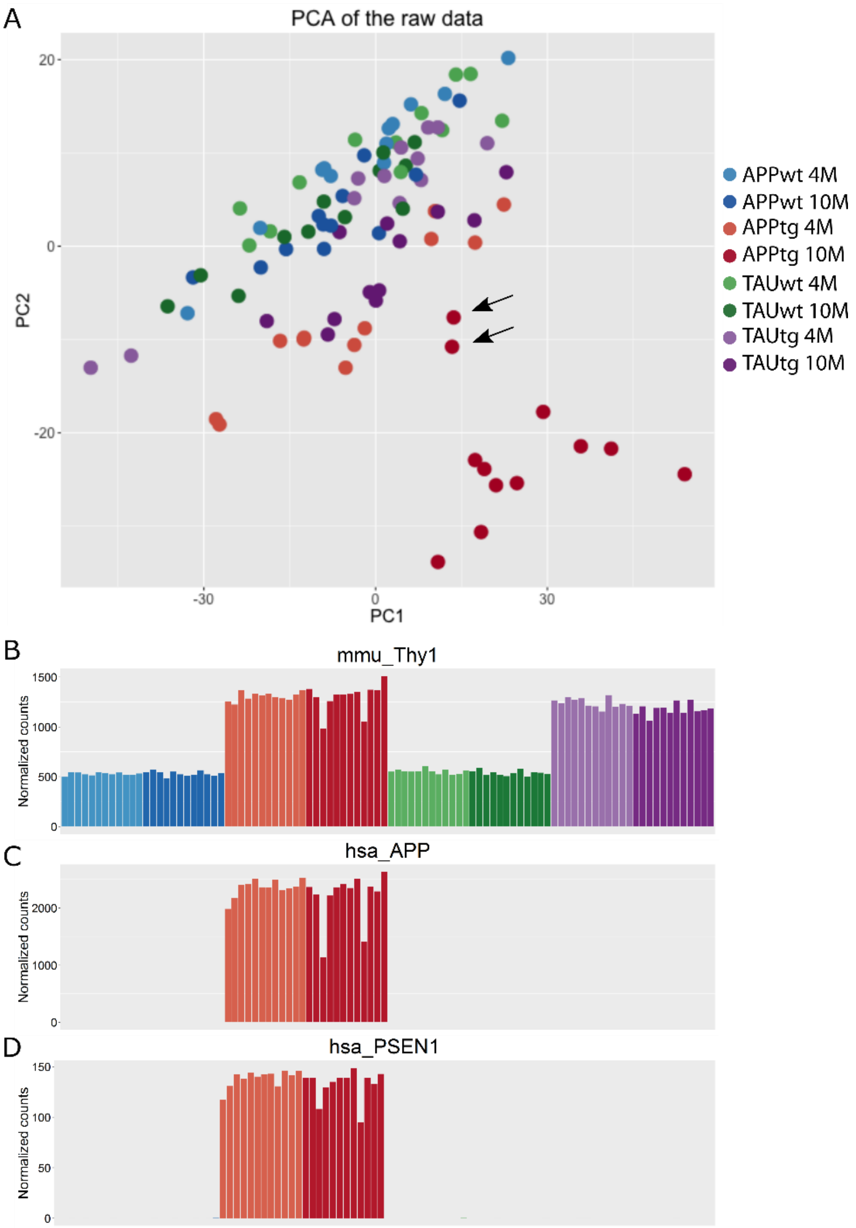

Appendix Fig.S1: Two APPtg-10M mice have lower transgene expression. A: The first 2 principle components of the PCA plot of the log2(raw reads from Feature Counts +1) demonstrate that 2 APPtg-10M mice cluster more towards the APPtg-4M mice (see arrows). B-D: These two mice have decreased expression of transgenes mmu-Thy1 (-24%; B), hsa-APP (-46%; C) and hsa-PSEN1 (-26%; D) compared

to other APPtg-10M mice. Expression of hsa-MAPT, mmu-App, mmu-Psen1 and mmu-Mapt remained unaffected (data not shown).

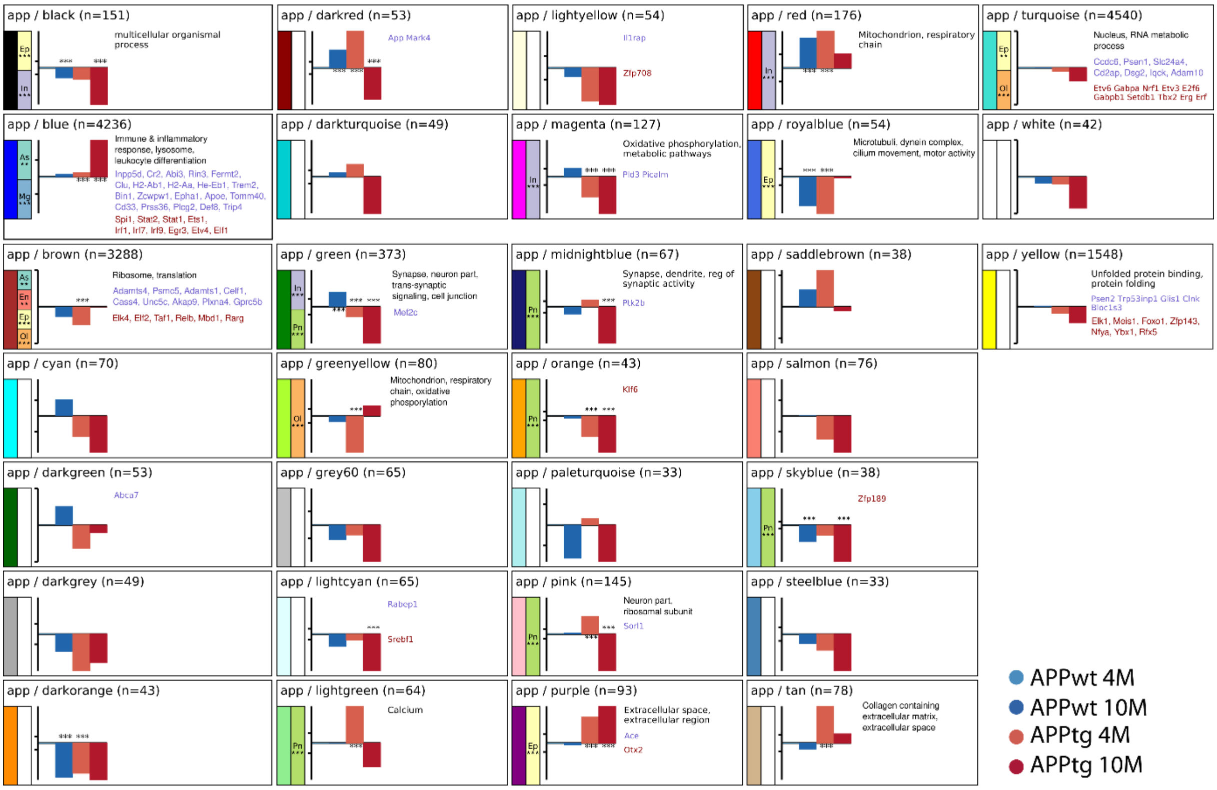

Appendix Fig.S2: Overview of APPtg-based WGCNA modules. For each module is indicated the z-score distribution of each experimental group and their bonferroni-adjusted empirically-derived p-values. If significant GO enrichment is observed, the categories are mentioned in the top row (see Datasets EV2 and EV3). If the module contains established GWAS genes, they are reported on the middle row (purple font). If there is an enrichment of transcription factor targets of transcription factors expressed in that module, the transcription factors are labeled on the bottom row (red font). If significant enrichment with a specific cell type gene set is observed, this is indicated with the cell-type labeled colored boxes. As: astrocytes; En: Endothelial; Ep: Ependymal; In: interneurons; Mg: microglia; Ol: oligodendrocyte; Pn: Pyramidal neuron.

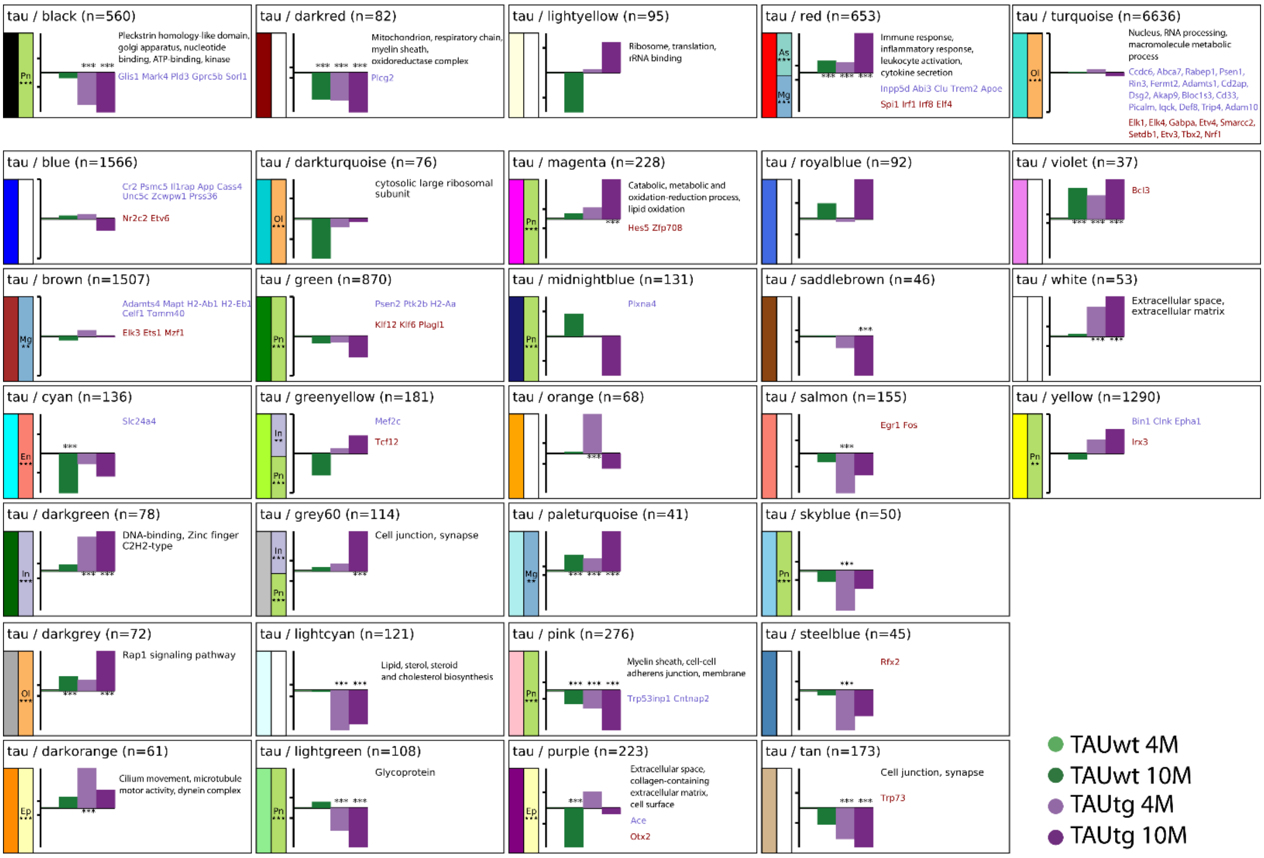

Appendix Fig.S3: Overview of TAUtg-based WGCNA modules. For each module is indicated the z-score distribution of each experimental group and their bonferroni-adjusted empirically-derived p-values. If significant GO enrichment is observed, the categories are mentioned in the top row (see Datasets EV2 and EV3). If the module contains established GWAS genes, they are reported on the middle row (purple font). If there is an enrichment of transcription factor targets of transcription factors expressed in that module, the transcription factors are labeled on the bottom row (red font). If significant enrichment with a specific cell type gene set is observed, this is indicated with the cell-type labeled colored boxes. As: astrocytes; En: Endothelial; Ep: Ependymal; In: interneurons; Mg: microglia; Ol: oligodendrocyte; Pn: Pyramidal neuron.

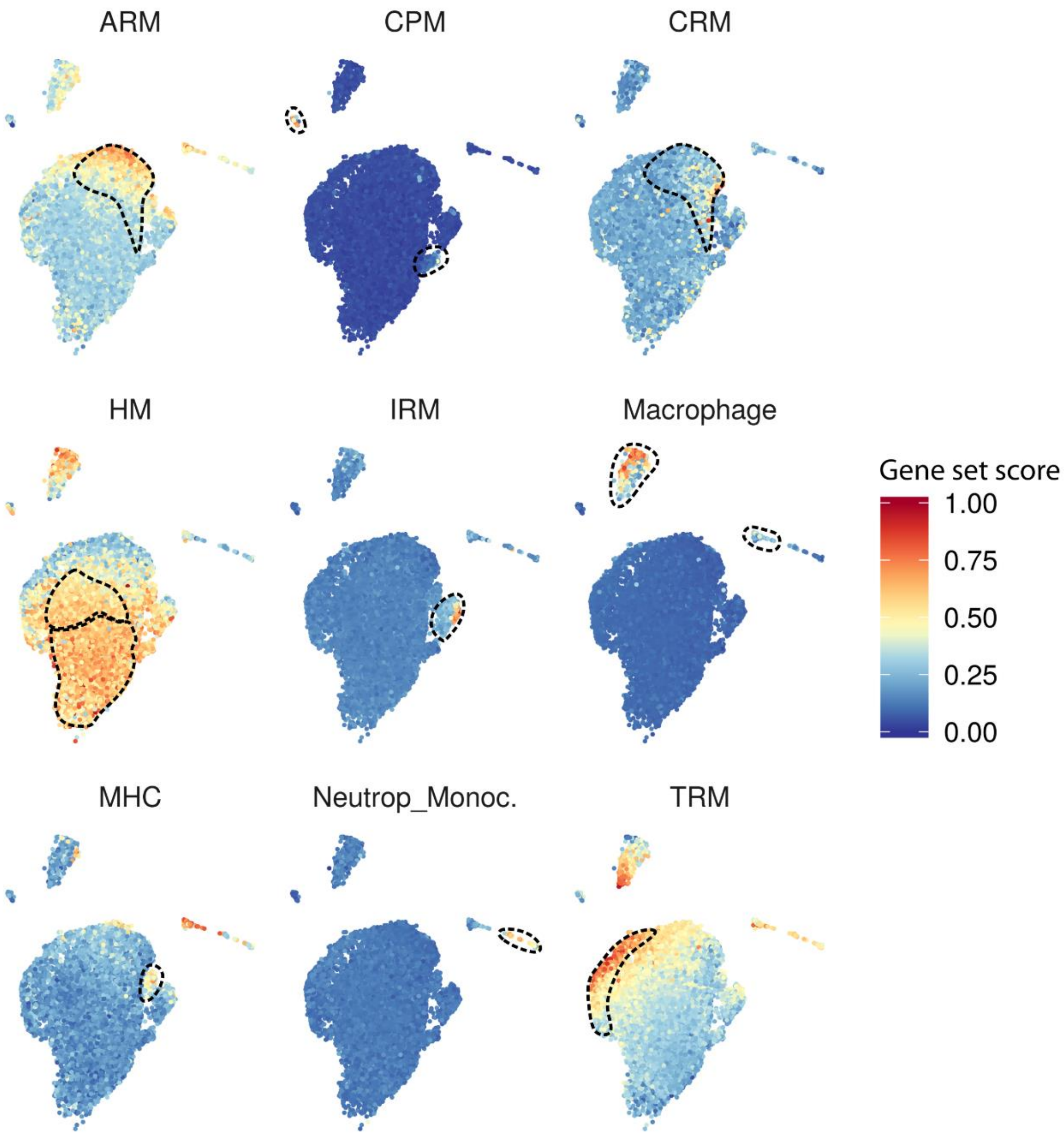

Appendix Fig.S4: Expression of myeloid marker gene sets across the UMAP of the single microglia sequencing data. UMAP plot as in Fig.6A, coloured by a score of the combined level of expression of publicly available gene sets that characterize distinct microglial states (ARM, CPM, HM, IRM and TRM from (Sala Frigerio *et al*, 2019); CRM from (Mancuso *et al*, 2019a) which was demonstrated to be a subset of ARM; Neutrophil-Monocytes and Macrophages from (Friedman *et al*, 2018); a list of MHC and CPM markers can be found in Dataset EV6). The original clusters from Fig.6A are demarcated. Scores are scaled from 0 to 1.

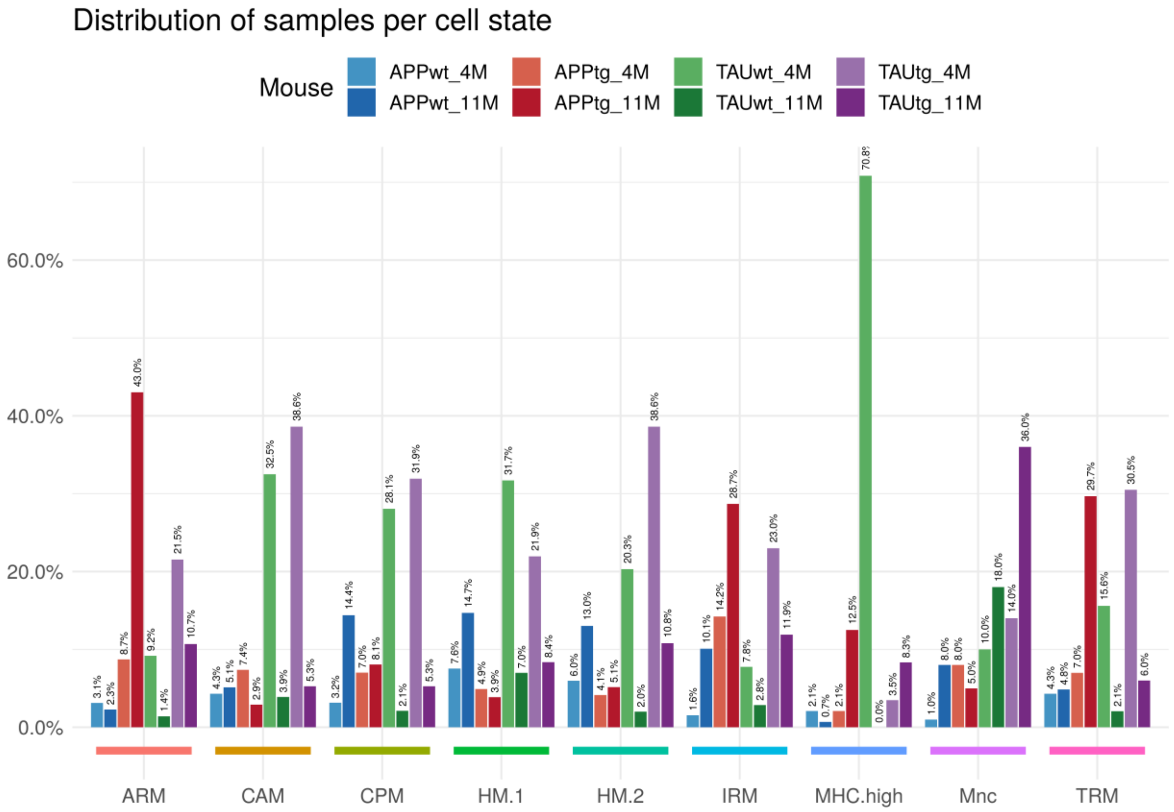

Appendix Fig.S5: Division of experimental groups within each cluster. Per cluster (100%) the percentage of cells belonging to each experimental group was calculated.

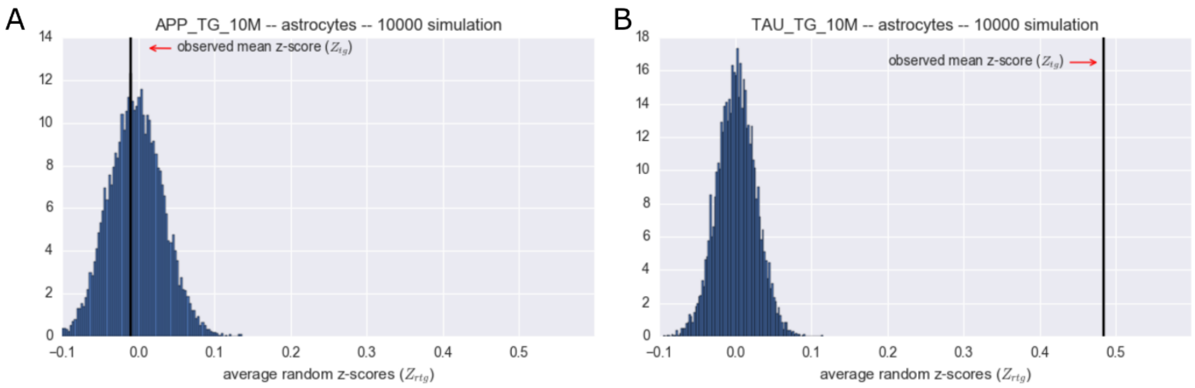

Appendix Fig.S6: Examples of the mean z-score distribution of 10,000 randomly sampled gene sets with equal sizes to the gene set of interest (e.g. 719 astrocyte genes). The black vertical line represents the observed mean z-score Z_{tg} for APP-TG-10M (A) or TAU-TG-10M mice (B). Its cumulated distribution function among the normally distributed random z-scores Z_{rtg} determines the associated empirical p-value.

Appendix Tables

Appendix Table S1

Overview of the number of Marioni-based GWAS genes at different p-value cutoffs. For each cutoff is listed the number of genes in the gene set (marioni), how many of these are expressed in mouse hippocampus, out of those how many are in the APPtg-Blue module (marioni_blue), how many are significantly differentially expressed (marioni_DE), how many are both in APPtg-Blue and significantly differentially expressed (marionin_DE_blue) and how many of those genes are 'new', i.e. not established AD GWAS genes

**p-value_cutoff**	**marioni**	**hippocampus_expressed**	**marioni_blue**	**marioni_DE**	**marioni_DE_blue**	**new**
0.5	9028	7274	1956	414	263	254
0.1	2738	2251	622	142	90	81
0.05	1799	1518	439	107	72	63
0.01	771	644	200	50	37	29
0.005	572	475	158	39	31	23
0.001	314	263	88	24	18	11
0.0005	255	215	72	19	15	8
0.0001	168	139	52	11	9	2
5.00E-05	146	120	47	10	9	2
1.00E-05	108	86	33	9	8	2
5.00E-06	92	74	26	8	7	2
1.00E-06	70	56	21	5	5	1
5.00E-07	66	52	20	5	5	1
1.00E-07	55	41	16	4	4	1

Appendix Table S2

Overlap, log2 odds ratio (LOR) & Benjamini-Yuketieli-adjusted p-value (BY_padj) between genes within a WGCNA module and the cell-type specific gene sets for astrocytes, endothelial, ependymal, interneurons, microglia, pyramidal neurons, and oligodendrocytes.

**Mouse_model**	**Module**	**Cell_type**	**LOR**	**overlap**	**genes_in_module**	**genes_in_celltype**	**BY_padj**
APPtg	black	ependymal	1.75	17	151	484	7.68E-06
APPtg	black	interneuron	2.12	18	151	365	2.10E-08
APPtg	blue	astrocyte	0.54	70	4236	240	0.014651559
APPtg	blue	microglia	1.90	264	4236	436	1.74E-77
APPtg	brown	astrocyte	0.54	56	3288	240	0.036129912
APPtg	brown	endothelial	0.46	77	3288	353	0.035032181
APPtg	brown	ependymal	0.54	112	3288	484	0.000214844
APPtg	brown	oligodendrocyte	0.55	106	3288	453	0.000226802
APPtg	green	interneuron	1.62	28	373	365	1.13E-08
APPtg	green	pyramidal	1.93	65	373	703	2.27E-26
APPtg	greenyellow	oligodendrocyte	2.33	14	80	453	2.15E-07
APPtg	lightgreen	pyramidal	1.73	10	64	703	0.003650091
APPtg	magenta	interneuron	1.63	10	127	365	0.005281205
APPtg	midnightblue	pyramidal	2.42	18	67	703	8.57E-10
APPtg	orange	pyramidal	2.08	9	43	703	0.000902328
APPtg	pink	pyramidal	1.65	21	145	703	1.69E-06
APPtg	purple	ependymal	2.32	17	93	484	6.79E-09
APPtg	red	endothelial	2.27	23	176	353	6.46E-12
APPtg	royalblue	ependymal	3.92	28	54	484	2.24E-29
APPtg	skyblue	pyramidal	2.09	8	38	703	0.002600416
APPtg	turquoise	ependymal	0.38	134	4540	484	0.014651559

APPtg	turquoise	oligodendrocyte	0.43	130	4540	453	0.003809476
TAUtg	black	pyramidal	1.28	57	560	703	7.64E-12
TAUtg	brown	microglia	0.55	49	1507	436	0.041686889
TAUtg	cyan	endothelial	1.70	11	136	353	0.001592094
TAUtg	darkgreen	interneuron	2.40	12	78	365	1.33E-06
TAUtg	darkgrey	oligodendrocyte	2.16	11	72	453	4.03E-05
TAUtg	darkorange	ependymal	4.09	34	61	484	3.05E-37
TAUtg	darkturquoise	oligodendrocyte	1.98	10	76	453	0.000471521
TAUtg	green	pyramidal	0.85	60	870	703	5.10E-06
TAUtg	greenyellow	interneuron	1.36	11	181	365	0.020015354
TAUtg	greenyellow	pyramidal	1.22	18	181	703	0.002622571
TAUtg	grey60	interneuron	1.96	12	114	365	6.99E-05
TAUtg	grey60	pyramidal	2.16	25	114	703	8.82E-12
TAUtg	lightgreen	pyramidal	2.01	21	108	703	9.15E-09
TAUtg	magenta	astrocyte	1.90	15	228	240	6.65E-06
TAUtg	midnightblue	pyramidal	1.58	18	131	703	3.58E-05
TAUtg	paleturquoise	microglia	2.14	6	41	436	0.013252102
TAUtg	pink	pyramidal	1.02	23	276	703	0.003612158
TAUtg	purple	ependymal	2.24	37	223	484	5.35E-19
TAUtg	red	astrocyte	1.10	20	653	240	0.003840311
TAUtg	red	microglia	2.06	78	653	436	5.33E-35
TAUtg	skyblue	pyramidal	2.03	10	50	703	0.000468148
TAUtg	turquoise	mural	0.61	69	6636	155	0.013252102
TAUtg	turquoise	oligodendrocyte	0.60	200	6636	453	9.16E-08
TAUtg	yellow	pyramidal	0.54	67	1290	703	0.007579955

Appendix Table S3

Results from the SuperExactTest (Wang et al. 2015) testing the statistical significance of finding overlaps between 2 or more data sets. For every dataset or combination of datasets it is given what the observed and expected overlaps are, what the fold enrichment is of the observed/expected overlap, the unadjusted p-value and the Benjamini-Yuketieli adjusted p-value.

**Intersected_gene_sets**	**Observed.Overlap**	**Expected.Overlap**	**Fold.enrichment**	**P.value**	**BY.padj**
APPblue & DEint	493	155.2	3.2	1.54E-158	1.28E-157
gwas & DEint	24	11.5	2.1	0.000597458	0.001244704
gwas & APPblue	88	61.1	1.4	0.000138617	0.000385047
gwas & APPblue & DEint	18	2.2	8.0	2.17E-11	9.04E-11
