# Supplementary material for: Novel Alzheimer risk genes determine the microglia response to amyloid‐β but not to TAU pathology
Source: EMBO Mol Med. 2020 Jan 17;12(3):e10606. doi: 10.15252/emmm.201910606 (PMC7059012; doi:10.15252/emmm.201910606)
Supplement: Supplementary file 2 — Expanded View Figures PDF [file EMMM-12-e10606-s002.pdf]

Expanded View Figures

Figure EV1. APPtg and TAUtg mice demonstrate divergent neuronal and glial responses to increasing pathology load.

Z-score distribution per experimental group for all genes in cell type-specific gene sets as defined by Zeisel *et al* (2015) and SynaptomeDB (Pirooznia *et al*, 2012). Boxplots: center line, median; box limits, 25th–75th quartiles; and whiskers, 1.5× interquartile range. Empirical *P*-values are Bonferroni-adjusted (P_{bonf}) and indicate significant shift in *z*-score distribution (see Materials and Methods). ***$P_{bonf} < 0.001$, **$P_{bonf} < 0.01$, *$P_{bonf} < 0.05$.

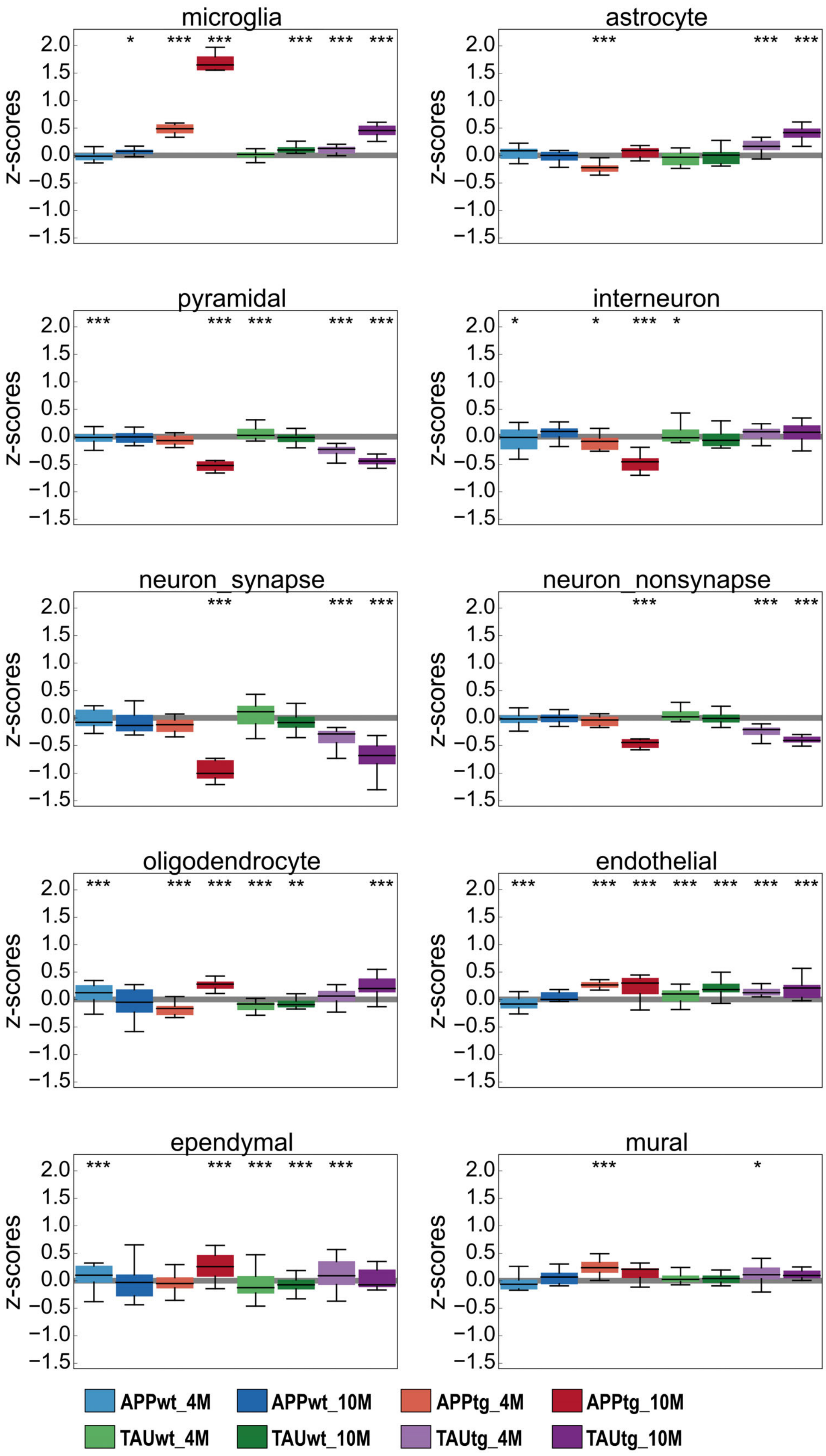

Figure EV1.

© 2020 The Authors

Figure EV2. Expression of the top 18 genes in single microglia RNA-seq and publicly available data.

A Expression of the top 18 genes in the bulk RNA-seq dataset across the 8 experimental groups. Boxplots: center line, median; box limits, 25th–75th quartiles; and whiskers, 1.5× interquartile range.
B Scaled expression of our top 18 genes in the single microglia RNA-seq dataset, highlighting that Clu, Gpc2, and Treml2 are very sparsely expressed by microglia. Expression values are scaled from 0 to 1. The insert on the far right is a duplicate of Fig 6A to help locate the expression of specific genes to particular clusters.
C Compilation of publicly available data and the bulk and single microglia sequencing from the current study. Whereas bulk RNA-seq of mouse models (rows 1–3) and AD patients (row 12–16) demonstrates increased expression in the top 18 transcripts, RNA-seq of isolated microglia (rows 8–11) or single cell RNA-seq (rows 4–7) demonstrates no alterations or slightly decreased expression of the top 18 genes.

© 2020 The Authors

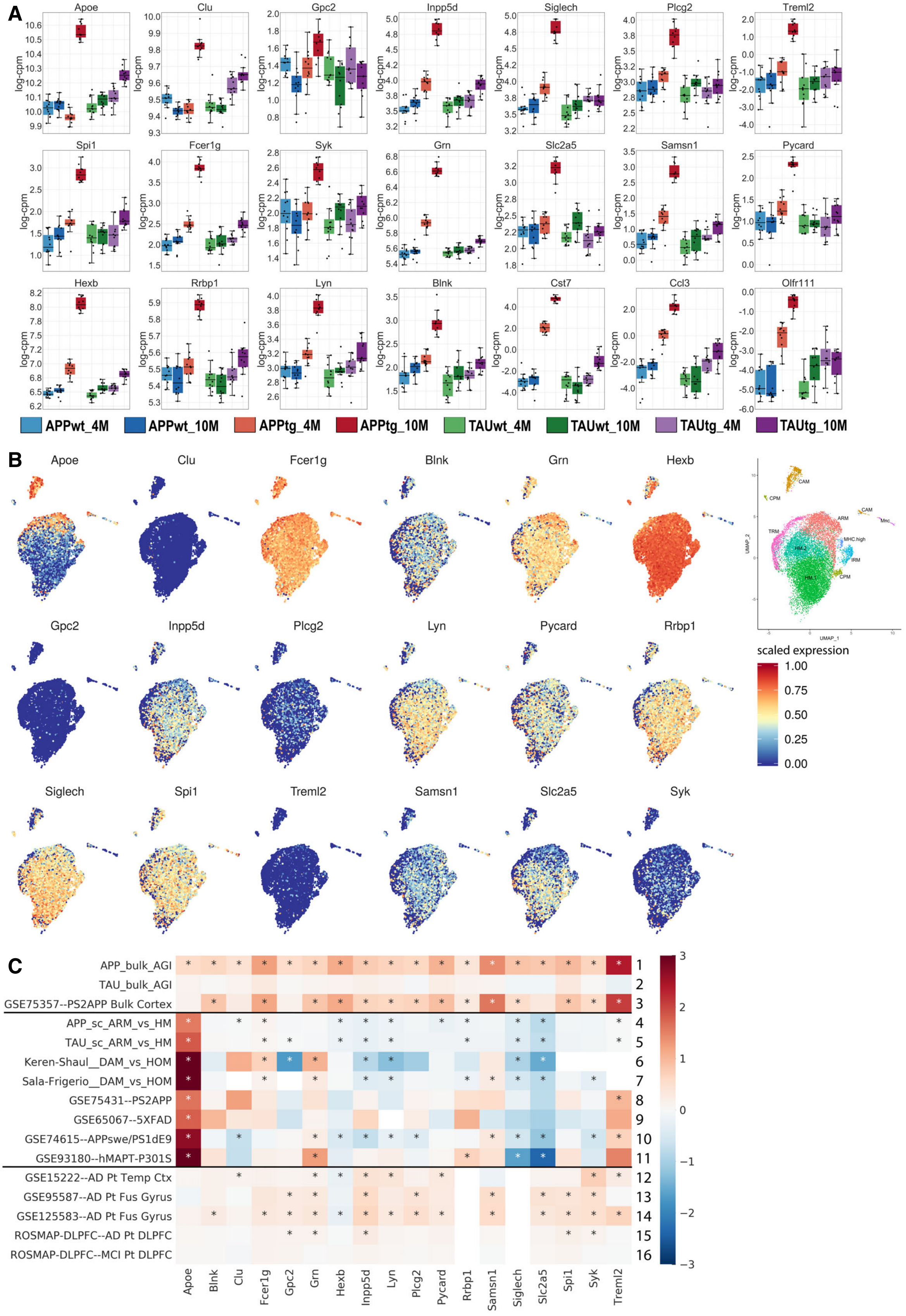

Figure EV2.

 © 2020 The Authors

Figure EV3. Examples of the predicted contribution of microglial expression of *Apoe* (A), *Clu* (B), and *Inpp5d* (C) to their observed expression in bulk.

A–C For each gene, the different panels display i) the average expression of the specified gene in the single cell microglia RNA-seq for all microglia, homeostatic microglia (HM), and non-homeostatic microglia (ARM); ii) the predicted level of microgliosis in each experimental group, based on the average expression of the microglial gene set of Zeisel *et al* (2015), divided in the contribution of ARM and HM cells to the predicted microgliosis. As this prediction is not gene-dependent, graph ii is the same in panels A, B, and C; iii) the predicted microglial expression per microglial subtype multiplied with the degree of microgliosis; iv) linear regression on the predicted microglial expression as calculated in iii) and the observed bulk RNA-seq expression; and v) the observed bulk RNA-seq expression (in blue) per experimental group plotted side-by-side with the predicted microglial expression (in green) of that specific gene.

© 2020 The Authors

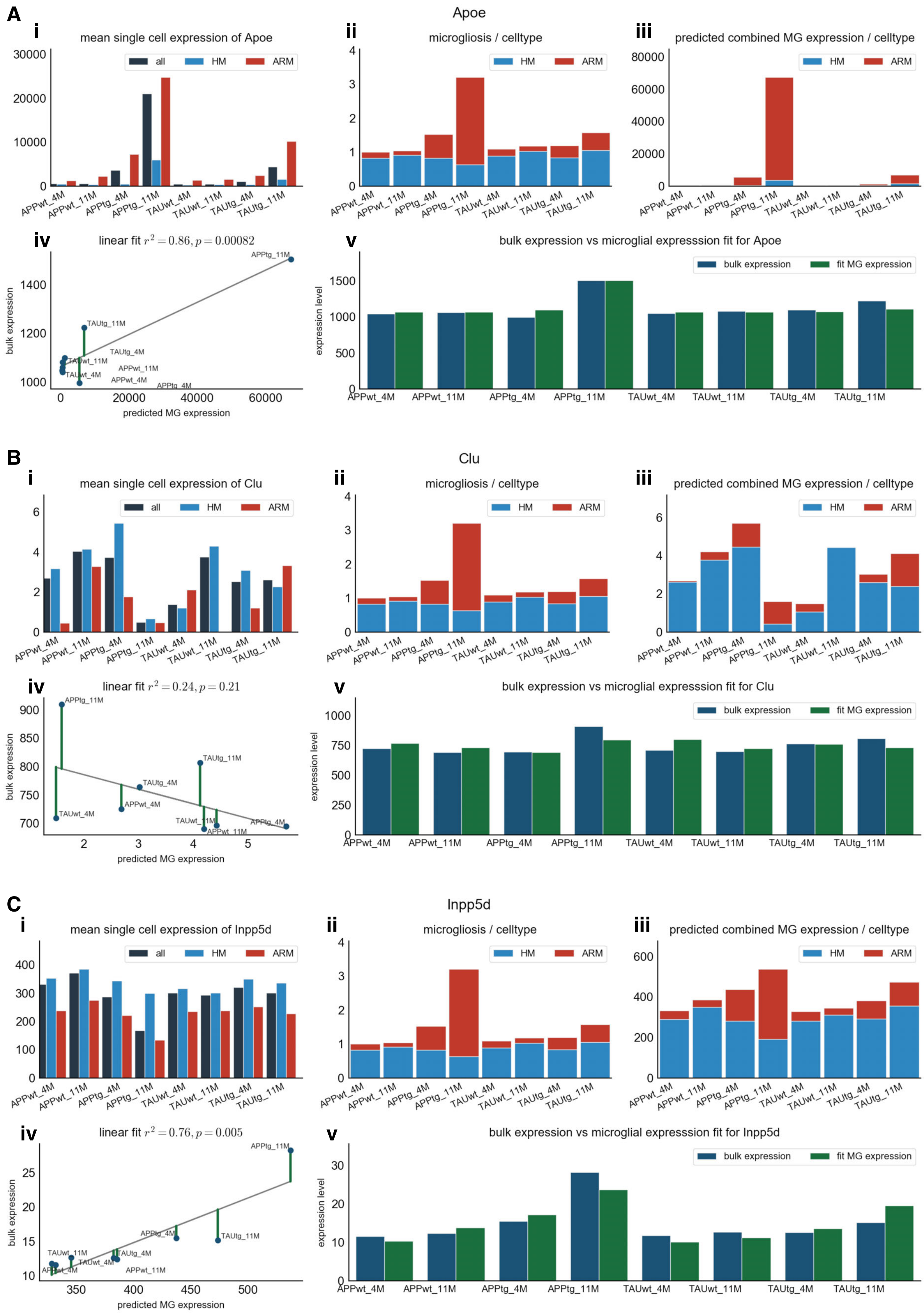

Figure EV3.

 © 2020 The Authors
